# Test-retest reliability of myelin imaging in the human spinal cord: Measurement errors versus region- and aging-induced variations

**DOI:** 10.1371/journal.pone.0189944

**Published:** 2018-01-02

**Authors:** Simon Lévy, Marie-Claude Guertin, Ali Khatibi, Aviv Mezer, Kristina Martinu, Jen-I Chen, Nikola Stikov, Pierre Rainville, Julien Cohen-Adad

**Affiliations:** 1 NeuroPoly Lab, Institute of Biomedical Engineering, Polytechnique Montreal, Montreal, QC, Canada; 2 Centre de Recherche de l'Institut Universitaire de Gériatrie de Montréal (CRIUGM), Montréal, QC, Canada; 3 Montreal Health Innovations Coordinating Center (MHICC), Montreal Heart Institute, Montreal, QC, Canada; 4 Psychology Department, Bilkent University, Ankara, Turkey; 5 Interdisciplinary program in Neuroscience, Bilkent University, Ankara, Turkey; 6 National Magnetic Resonance Research Center (UMRAM), Bilkent University, Ankara, Turkey; 7 The Edmond and Lily Safra Center for Brain Sciences (ELSC), The Hebrew University of Jerusalem, Jerusalem, Israel; 8 Department of Stomatology, Faculty of Dentistry, Université de Montréal, Montreal, QC, Canada; 9 Montreal Heart Institute, Montreal, QC, Canada; 10 Functional Neuroimaging Unit, CRIUGM, Université de Montréal, Montreal, QC, Canada; Instituto Cajal-CSIC, SPAIN

## Abstract

**Purpose:**

To implement a statistical framework for assessing the precision of several quantitative MRI metrics sensitive to myelin in the human spinal cord: T_1_, Magnetization Transfer Ratio (MTR), saturation imposed by an off-resonance pulse (MT_sat_) and Macromolecular Tissue Volume (MTV).

**Methods:**

Thirty-three healthy subjects within two age groups (young, elderly) were scanned at 3T. Among them, 16 underwent the protocol twice to assess repeatability. Statistical reliability indexes such as the Minimal Detectable Change (MDC) were compared across metrics quantified within different cervical levels and white matter (WM) sub-regions. The differences between pathways and age groups were quantified and interpreted in context of the test-retest repeatability of the measurements.

**Results:**

The MDC was respectively 105.7ms, 2.77%, 0.37% and 4.08% for T_1_, MTR, MT_sat_ and MTV when quantified over all WM, while the standard-deviation across subjects was 70.5ms, 1.34%, 0.20% and 2.44%. Even though particular WM regions did exhibit significant differences, these differences were on the same order as test-retest errors. No significant difference was found between age groups for all metrics.

**Conclusion:**

While T_1_-based metrics (T_1_ and MTV) exhibited better reliability than MT-based measurements (MTR and MT_sat_), the observed differences between subjects or WM regions were comparable to (and often smaller than) the MDC. This makes it difficult to determine if observed changes are due to variations in myelin content, or simply due to measurement error. Measurement error remains a challenge in spinal cord myelin imaging, but this study provides statistical guidelines to standardize the field and make it possible to conduct large-scale multi-center studies.

## 1. Introduction

### 1.1. Quantitative MRI

Precise techniques are needed to monitor microstructural degeneration of the nervous tissue in clinics, especially for longitudinal follow up of white matter (WM) lesions in neurodegenerative pathologies, such as demyelination in multiple sclerosis. Rather than using MRI as a technique for simply viewing the anatomy, quantitative MRI (qMRI) aims to provide quantitative *metrics* related to some tissue properties. To date, several qMRI metrics have been proposed to characterize myelin content in the WM.

The longitudinal relaxation time T_1_ has shown high correlation with the myelin volume quantified by histology [1–3]. However, T_1_ is also affected by iron concentration [4], and it is difficult to disentangle the specific contribution of myelin and iron because of their co-localization [5]. The Magnetization Transfer Ratio (MTR) has also shown high correlation with histopathology of myelin in multiple sclerosis patients [2,3]. However, MTR consists of various contributions (T_1_ and fraction F of exchanging protons bound to macromolecules) [6,7], which in some cases work against each other, reducing its sensitivity to myelin [2,8]. In this perspective, the quantification of the saturation imposed by an off-resonance pulse (MT_sat_) has been proposed to minimize T_1_ effects and increase the specificity to myelin [6].

Proton density (PD) is also a promising metric, as it measures the density of MRI-visible protons–i.e. protons with sufficiently long transversal relaxation time (T_2_)–which are water (or liquid) protons. In the Central Nervous System (CNS), the complement of PD yields an estimate of the density of non-free protons, which are mostly bound to lipids and other macromolecules. Since myelin consists of 70 to 80% lipids and some macromolecules [9,10], this index can be expected to be a good marker of myelin content. Several PD estimation techniques and studies in the CNS have been published [11–23]. The complement of PD has been recently named *Macromolecular Tissue Volume* (MTV) [24,25] and its sensitivity and specificity to myelination was tested. MTV showed high accuracy and precision when quantifying the lipid content in phantoms. In addition, the MTV significantly decreased in the WM of multiple sclerosis patients compared to controls, showing independence from fiber geometry, unlike the Fractional Anisotropy (FA) from Diffusion Tensor Imaging (DTI). However, since MTV is defined as the fraction of non-liquid protons, it includes more than the volume occupied by myelin, raising the question of its specificity to myelin.

Myelin Water Imaging (MWI) using multi-echo T_2_ [26] is another myelin mapping technique that has shown good sensitivity to myelin content in MS patients *post-mortem* [27] and *in vivo* [28]. While the earliest implementations of MWI were not clinically feasible, techniques such as Gradient- And Spin-Echo (GRASE [29,30]) were shown to speed up the acquisition [31]. Further investigations are ongoing.

The time constant of the transverse relaxation due to spin-spin interactions and local field inhomogeneities (T_2_*) has also exhibited sensitivity to myelin [32–34]. However, T_2_* includes important contributions from other factors, such as iron content [4,35], fiber orientation [36], blood vessels [37] and blood oxygen level [38].

Inhomogeneous Magnetization Transfer (ihMT) ratio is another recent metric [39] that is thought to be particularly sensitive and specific to myelin [40,41]. However, the measurement of this metric requires non-product sequence which are currently not available on clinical scanners.

### 1.2. Terminology

The above-mentioned metrics have their own advantages and limitations in quantifying myelin content in the CNS. To compare them, the relevant criteria for a myelin biomarker needs to be defined properly. *Sensitivity* and *specificity* are often the outstanding criteria. Here, sensitivity refers to the ability of the metric to monitor the variations in myelin content, while the specificity describes its exclusivity to myelin variations, i.e. to what extent the variations in the metric values are due to variations in the myelin content *only*. However, before tackling the sensitivity and specificity of a metric, it is essential to assess its *repeatability*. Indeed, sensitivity and specificity cannot be determined precisely if the metric values dramatically change between different scan sessions. The repeatability refers to the agreement (measurement precision) between two or more measurements made at different time points under the same conditions (e.g., same protocol, same scanner, same subjects, etc.) [42]. The repeatability must not be mistaken with *reproducibility*, which refers to the agreement between two or more measurements made at different time points under changing conditions. In both repeatability and reproducibility studies, the *reliability* is a relevant aspect to assess. The reliability compares the variability of scores due to measurement errors to the variability in the “true”, error-free scores, i.e. to the variability induced by true variations of the measured feature (e.g., true variations in myelin content).

### 1.3. Review of past studies on qMRI metrics repeatability

The question of repeatability is even more relevant for spinal cord studies, where noise, motion and susceptibility artifacts make it difficult to acquire high quality images [43]. Previous studies investigated the repeatability of quantitative MRI metrics. Taso et al. [44] reported the repeatability of MTR, ihMTR and DTI (Diffusion Tensor Imaging) indexes within 3 healthy subjects at 3 time points by means of coefficients of variations (CV), defined as the ratio of the between-scans standard-deviation over the mean across scans. However, this index does not allow to properly compare between different metrics, as the means can differ drastically across metrics or even for a single metric across different studies (e.g., MTR [45]), yielding lower CVs for metrics with higher mean values. Smith et al. [46] also reported the test-retest repeatability of DTI and MT metrics within 9 healthy subjects at 2 time points using the normalized Bland-Altman difference (i.e. mean difference between scans divided by the mean across scans), which makes it harder to compare the repeatability between metrics with different means. Grussu et al. [47] reported the test-retest repeatability of NODDI (Neurite Orientation Dispersion and Density Imaging) indexes within 5 heathy subjects. The test-retest reliability was quantified by means of Intra-Class Correlation (ICC) coefficients defined as the ratio of the inter-subject variance over the total variance (i.e. the sum of the within- and between-subjects variances). Smith et al. [48] assessed the repeatability of MTR and F (fraction of exchanging protons bound to macromolecules) from quantitative magnetization transfer (qMT) imaging by means of the 95% confidence interval for the test-retest difference. However, this estimate of the measurement error was not properly compared neither between metrics nor in the context of the differences observed between (expected) different myelin contents.

The test-retest repeatability has been studied extensively in research fields other than qMRI, notably in rehabilitation research [49–53]. Useful statistical indexes to quantify repeatability are provided. First, the existence of a systematic bias between test and retest measurements can be examined by the confidence interval for the test-retest difference (*CI*_*d*_), as used in Smith et al. [48]. Then, the reliability can be assessed by the intra-class coefficient based on a two-way mixed effects model of analysis of variance. Finally, groups can be compared taking measurement errors into account (which is not done with usual statistical tests) using *CI*_*d*_, showing whether the difference between groups is distinguishable from measurement errors or not. In the same vein, one can compute the Minimum Detectable Change (MDC) to quantify the minimum difference between two single metric values that is necessary to report a “true” error-free change, again taking the measurement errors into account. The MDC is particularly appropriate and intuitive for clinicians who would like to assess whether a treatment affects their patient or not.

In this work, we propose a statistical framework to quantify the test-retest reliability of qMRI metrics. We *(i)* quantify the repeatability of T_1_, MTR, MT_sat_ and MTV in the spinal cord using a clinically-compatible protocol and *(ii)* evaluate the sensitivity of these metrics to myelin content across spinal pathways and age groups, in the context of the test-retest measurement errors.

## 2. Material and methods

### 2.1. Data acquisition

Thirty-three right-handed healthy subjects including 19 young (aged 24.9 ± 3.9, from 21 to 33 y.o.; 9 women, 10 men) and 14 elderly (aged 67.4 ± 4.0, from 61 to 73 y.o.; 6 women, 8 men) were recruited. A written consent form was obtained from each participant as supervised by the ethical review board of the Research Center of Montreal University Geriatric Institute (Comité mixte d’éthique de la recherche du RNQ, approval number CMER-RNQ_14-15-010).

To assess the metrics repeatability, 8 young (aged 24.0 ± 3.9, from 21 to 31 y.o., 2 women, 6 men) and 8 elderly (aged 67 ± 4.5, from 61 to 72 y.o., 2 women, 6 men) subjects from the previously described cohort underwent two scanning sessions: 12 subjects were scanned twice within a 10-month interval, and 4 within the same session (with a 5-minute break out of the scanner between scan and rescan). All data were acquired on a 3T Siemens TIM TRIO scanner and with a standard 12-channels head coil and a standard 4-channels neck coil.

The protocol consisted of:

- One sagittal turbo-spin-echo 3D SPACE T_2_-weigthed anatomic image (TR = 1500 ms; TE = 119 ms; flip angle = 120°; BW = 723 Hz/voxel; matrix = 384x384x52; resolution = 1x1x1 mm; FOV = 384x384x52 mm) with a high contrast between cord and cerebrospinal fluid (CSF) to further take the curvature of the cord into account in the data processing;
- Four 3D FLASH acquisitions (TR = 35 ms; TE = 5.92 ms; BW = 260 Hz/voxel; matrix = 192x192x22; resolution = 0.9x0.9x5 mm; gap = 1 mm; FOV = 174x174x110 mm; R = 2 acceleration; phase encoding direction = right-left). The four FLASH scans consisted of:
  ○ One with a prior RF saturation pulse (Gaussian-shaped, duration = 9984 μs, offset frequency = 1.2 kHz) and an excitation flip angle of 10°;
  ○ Three without a saturation pulse and flip angles of 4°, 10°, and 20°;
- Two axial 2D segmented spin-echo EPI acquisitions (TR = 3000 ms; TE = 19 ms; BW = 1905 Hz/voxel; matrix = 64x64, 17 slices; resolution = 3.0x3.0x5.5 mm; FOV = 192x192 mm) with a flip angle of 60 and 120° respectively (for B_1_^+^ estimation purposes);

All images spanned at least C2 to C5 vertebral bodies. The duration of the protocol was 18 minutes.

### 2.2. Data processing

Analysis was performed using the *Spinal Cord Toolbox* (SCT) version 2.2.3 [54]. The four datasets were first co-registered, then metrics were calculated. For extracting metrics within specific pathways in the white matter (dorsal column, DC, lateral funiculi, LF, ventral funiculi, VF), data were registered to the MNI-Poly-AMU template [55], which includes an atlas of WM tracts [56]. For sake of clarity, details about the processing pipeline are included in the supplementary material (see S1 File in section 8. Supporting information).

### 2.3. Statistical analysis

Statistical analyses were performed using MATLAB R2014a (The MathWorks, Inc., Natick, Massachusetts, USA) and SPSS (IBM SPSS Statistics–Release 24.0.0.0) at the 0.05 significance level unless otherwise stated.

#### 2.3.1. Repeatability

Systematic change between test and retest

The mean of the difference between test and retest $\left(\bar{d}\right)$ across subjects was computed along with a 95% confidence interval for the true test-retest difference (*CI*_*d*_) derived according to:

$${CI}_{d}=\bar{d}\pm t_{n-1}\cdot SE$$

where $SE={SD}_{d}/\sqrt{n}$ is the Standard Error, *SD*_*d*_ is the standard-deviation (SD) of the difference between test and retest across the subjects, *n* is the number of subjects and *t*_*n*−1_ is the *t* statistics with *n* − 1 degrees of freedom and type I error of 5% [57]. In our case, *t*_*n*−1_ = 2.131.

If zero is not included in *CI*_*d*_, we can consider that a systematic change between test and retest has occurred [50]. In addition to assess the systematic bias between test and retest, the CI_d_ gives the minimum difference between two subjects groups that is distinguishable from measurement errors.

Absolute test-retest difference

The absolute difference between test and retest, termed |*d*|, and its mean across subjects ($\bar{\left|d\right|}$) were computed to give to the reader a basic and direct measure of the measurement errors magnitude.

Reliability

The Intra-Class Correlation (ICC) coefficient is an appropriate coefficient to assess the test-retest reliability [58]. It measures the proportion of variance that is attributable to the “true” error-free scores of subjects (inter-subject variance) compared to the total variance (“true” variance + variance due to measurement errors). The ICC is calculated from a 2-way mixed effects model of repeated-measures analysis of variance which particularly fits any kind of test-retest experiment designs: the total variance is partitioned between within- and between-objects (subjects) variances. A commonly used index to report repeatability is the Pearson’s correlation coefficient. The ICC coefficient value is often close to the Pearson’s correlation value. However, the ICC includes a penalization for a systematic error between measurements (in this case, the ICC would be lower than the Pearson’s) and it can also assess the reliability of a measure based on more than two measurements by subjects (thanks to the model of analysis of variance used for computation). Moreover, the Pearson’s coefficient normalizes each measurement by its own mean and SD, whereas the ICC normalizes the variables by the pooled mean and SD of both measurements. So if the variables do not have a common unit and variance, the Pearson’s is more appropriate. But, for test-retest measurements having the same units, the ICC is a better index [59].

The higher the ICC, the higher the reliability; the upper threshold above which the ICC would reflect a good reliability remains subjective and depends on the application but we can still refer to the scale proposed by Shrout and Fleiss [58], Fleiss [60] and Cicchetti [61]: poor < 0.4 < fair < 0.6 < good < 0.75 < excellent ≤ 1. Chinn [62] suggests that measure needs to have at least an ICC coefficient of 0.6 to be useful. Contrary to the other repeatability indexes of this section, the ICC coefficient is a dimensionless index.

In this study, the ICC coefficient was computed according to the Matlab implementation of McGraw and Wong [59] (case 3A).

Minimal Detectable Change

Another useful index is the Minimal Detectable Change (MDC). It estimates the minimal difference between two scores that would reflect a “true” difference (i.e., not completely due to measurement error). It can be derived according to:

$$MDC=1.96\sqrt{2}\cdot SEM$$

where $SEM={SD}_{pooled}\sqrt{1-ICC}$ is the Standard Error of Measurement and *SD*_*pooled*_ is the standard-deviation across all measurements [49,63]. The MDC can also be interpreted as an interval for repeated measures. If *x* is the score of a subject for a single measurement, there is a 95% chance that the score of a repeated measurement lies within *x* ± *MDC*, assuming that the measurement errors are normally distributed. Any difference of ± MDC between two metric values can be considered as usual variation (due to measurement error); such a difference is not exceptional enough to be considered as a real change in the microstructure.

The MDC and the *CI*_*d*_ are based on the same idea of estimating the magnitude of the difference in metric values that can be only due to measurement errors. However, the MDC applies for two single metric values whereas *CI*_*d*_, which takes into account the sign of the difference between test and retest, applies for group comparison where negative measurement errors compensate for positive ones.

Comparison of indexes with different units across studies

To allow the comparison between techniques having different measuring units, one can express the repeatability indexes as a percentage of the mean across all measures, similar to calculation of the coefficient of variation (*CV* = 100 ∙ *SD*/*mean*). This method works fine when the mean is similar between techniques, otherwise the comparison is biased by the mean. For example, it has been shown that MTR could lead to drastically different mean values when acquired with different offset saturation pulse parameters, e.g. from 9 to 51% in the healthy WM [45]. Hence, normalizing by the mean would yield lower indexes for techniques with higher mean value, whereas these techniques could have the same test-retest repeatability as other techniques with lower mean values. To avoid this while still being able to compare between techniques side by side, we expressed these reliability indexes as a percentage of the SD across subjects of the first MRI session values only (*SD*_*subjects*_), i.e.:

$${Index}_{\%\;of\;{SD}_{subjects}}=100\cdot \frac{Index}{{SD}_{subjects}}$$

where *Index* represents any reliability index expressed in the metric unit such as the MDC. Indeed, this manipulation enables us to compare metrics side by side while accounting for the property we are looking for. Here, we are looking for a metric that has low test-retest variability relative to the inter-subject variability, i.e. relative to the dispersion of the sample this metric can offer. The SD across subjects is the most basic measure of the sample dispersion. In this way, we would like the ${Index}_{\%\;of\;{SD}_{subjects}}$ to be as low as possible (i.e., a low measurement error and a high SD across subjects) in order to observe differences between subjects that are higher than measurement errors.

#### 2.3.2. Sensitivity to myelin content variations

To assess the metrics sensitivity to the variations in myelin content across vertebral levels/WM regions relative to the repeatability, differences in group mean (*n* = 33) between levels/regions were compared along with their measurement error (assessed by the *CI*_*d*_).

Moreover, a one-way repeated measures ANOVA between levels/regions was performed independently for each metric (*n* = 33). The assumptions of normal distribution within each group (i.e., level or WM region) and of sphericity were checked using Lilliefors’s test and Mauchly's test respectively. When the assumption of sphericity was not met, a Greenhouse-Geisser correction was used to compute the ANOVA. When the ANOVA detected a significant difference, a post hoc multiple comparison test using the Tukey's honestly significant difference criterion was performed in order to find which groups were significantly different from each other.

To test the metrics sensitivity to the demyelination with aging reported by histology in the literature [64–66], for each vertebral level/WM region, means across each age group were compared taking the measurement error (assessed by the *CI*_*d*_ from the previous analysis) into account in order to investigate whether the difference in means could reflect a “true” difference or whether it is indistinguishable from measurement errors.

In addition, to test for significant differences, we performed independently for each metric, on the larger sample (*n* = 33, *n*_*young*_ = 19, *n*_*elderly*_ = 14), two-way repeated ANOVAs with the age group as between-subjects factor and, as within-subjects factor:

- vertebral levels to determine if this effect was consistent across levels (the metric being quantified in the whole WM);
- ROIs (WM, DC, LF, VF) to determine if this effect was consistent across ROIs (the metric being quantified from C2 to C4).

Finally, to complete this study, a power analysis was performed for two-tailed *t*-tests between young and elderly subjects based on whole WM values of each metric.

## 3. Results

### 3.1. Repeatability

**Fig 1:**
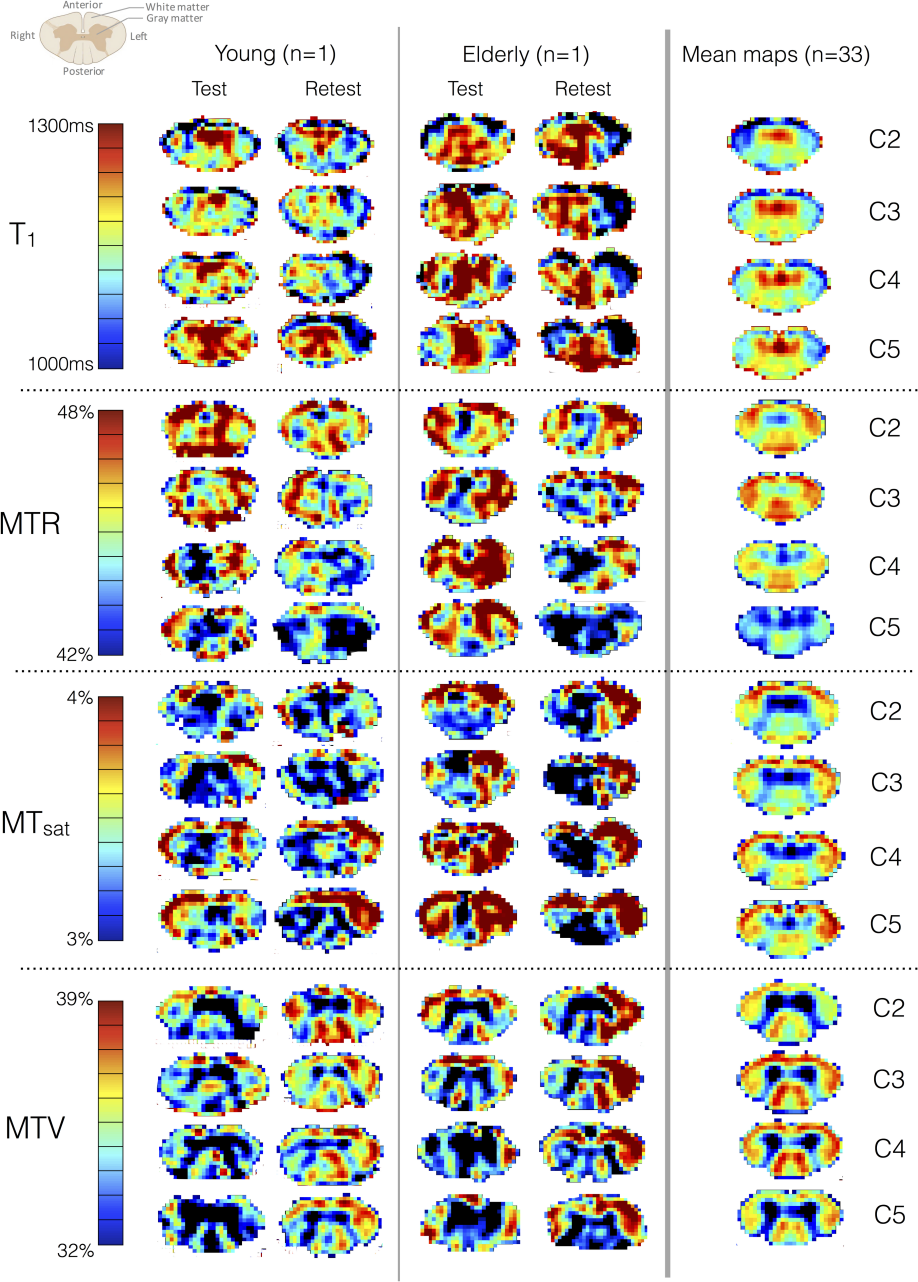
Test and retest maps in a young and an elderly subject at each vertebral level (mean across levels) along with the mean maps across the 33 subjects. All these maps are in the template space. Note that the color bar scale has been adjusted to the mean maps contrast. On a single-basis subject, one can observe a somewhat poor test-retest repeatability, within and across slices. However, despite this poor repeatability, the average maps (here, n = 33) are more consistent in terms of symmetry and tract-specific variations. For example, we can clearly distinguish higher MTV in the fasciculus cuneatus versus in the gracilis (dorsal column), which is in agreement with previous histology work [1,67].

Fig 1 shows test and retest multi-parametric maps by vertebral levels, for one single young and one single elderly subject, as well as for the group average (n = 33). The single subject data look noisy, however the average map shows clear distinction between WM and GM. Moreover, the symmetry that can be observed on the group average maps suggests no apparent differences in myelin content between left and right cord. In all metrics, the heterogeneity of values across WM regions suggests different microstructural compositions. For example, the fasciculus cuneatus shows higher MTV than the fasciculus gracilis, suggesting higher myelin content in agreement with previous histology studies [1,67]. Apart from MTR, all metrics show fairly stable values across vertebral levels.

#### A guide for reading (and understanding) figures and tables in the paper

**Fig 2:**
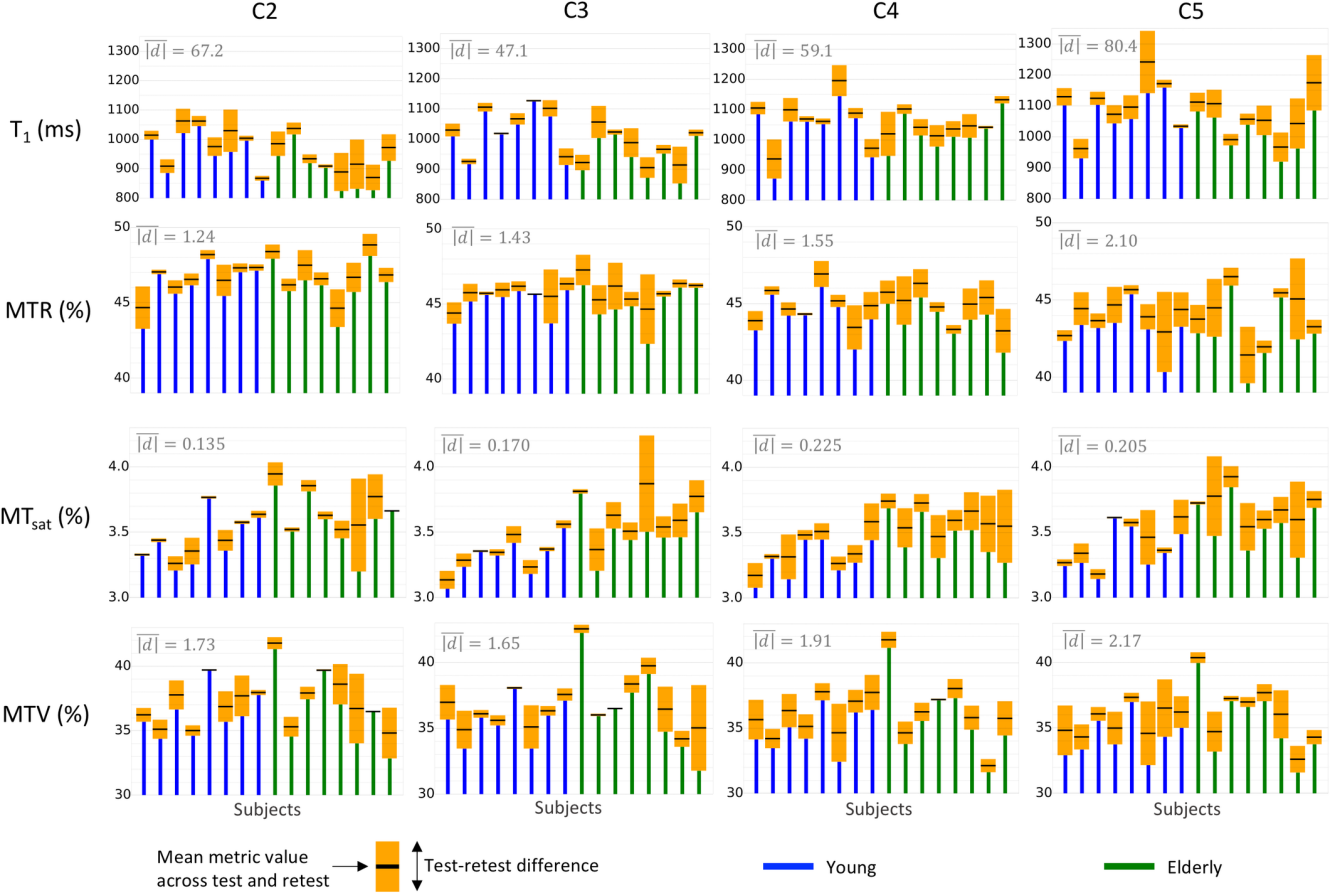
Subjects’ distribution with test-retest differences quantified over all WM according to vertebral levels. The top and bottom of the orange boxes respectively represent the max and min among test and retest, while the black line in the middle of the box represents the mean. Note that the y-axis does not start from zero for the sake of clarity. The mean absolute difference between test and retest (mean height of orange boxes, $\bar{\left|d\right|}$) is displayed in the top left hand corner of each graph. This figure gives a comprehensive view of the repeatability compared to between-subject differences.

**Fig 3:**
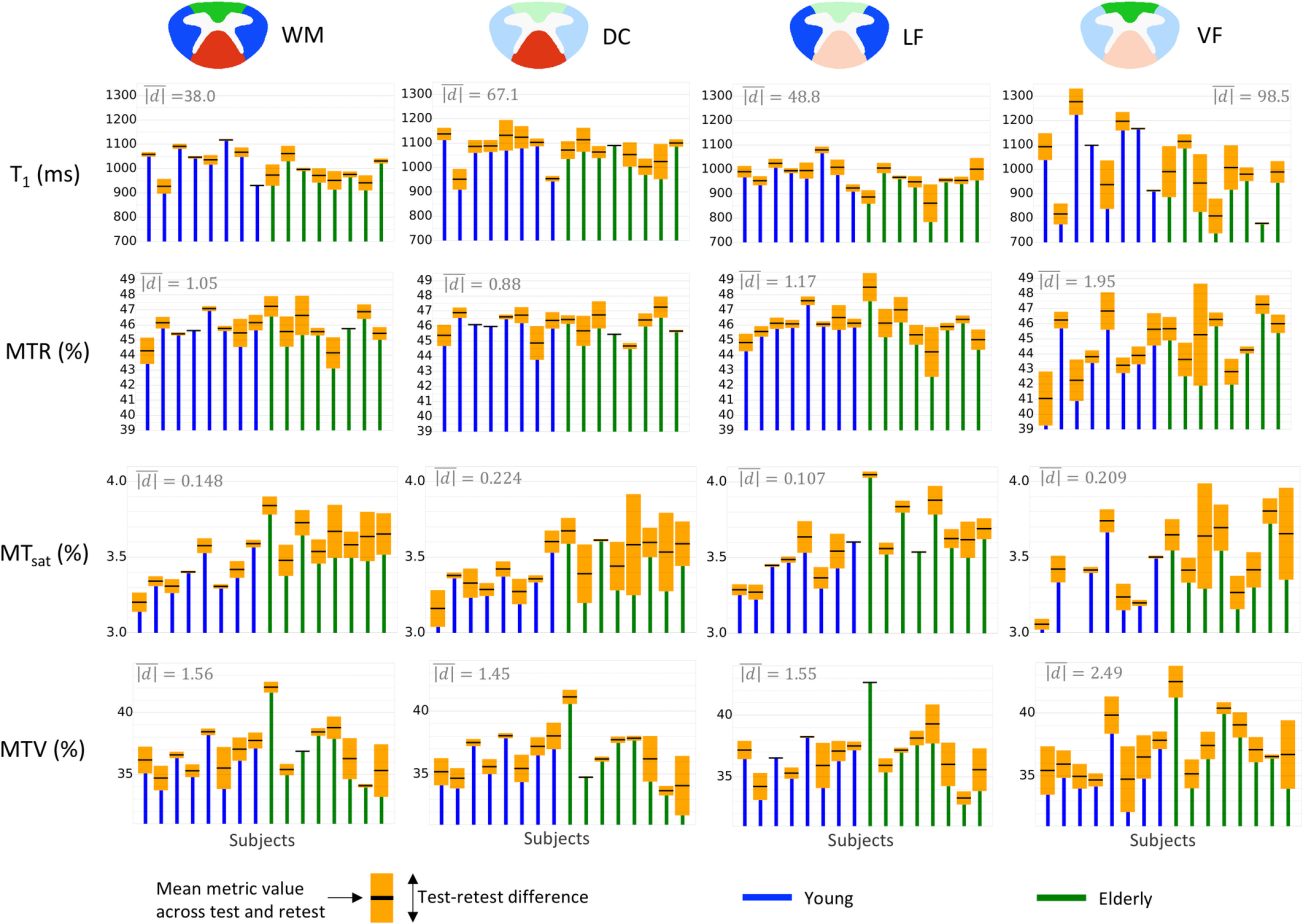
Subjects’ distribution along with the test-retest difference for each metric in the four ROIs. The top and bottom of the orange boxes are respectively the max and min among test and retest, while the black line in the middle of the box is the mean. The mean absolute test-retest difference (mean height of orange boxes, $\bar{\left|d\right|}$) across subjects is displayed in the top left hand corner of each graph. Due to its tiny size and its border location between GM and CSF, the VF yields the largest test-retest variations.

Fig 2 shows intra- and inter-subject differences for metrics quantified in the WM. Fig 2 is a subset of Table 1, which quantifies the metrics repeatability over all WM at the different cervical levels (Fig 3 and Table 2 are their analogs quantifying the metrics repeatability over all reliable levels within the different WM sub-regions). Let’s take an example to better explain how to use these repeatability indexes. Let’s take the T_1_ at C3. Regarding only one scan, the mean T_1_ across the group is 1007.2ms and the SD is 74.3ms. A 95% confidence interval for the mean test-retest difference of [-38.5; 23.1]ms indicates that if we rescan the same group a second time, the mean is likely to lie between 968.7 and 1030.3ms (with 95% probability). Now, if we measure T_1_ at C3 in a different group (e.g., a group of patients) and the resulting mean lies between 968.7 and 1030.3ms, we will not be able to report whether the difference in T_1_ between the two groups is due to measurement errors or to a true difference in T_1_. The MDC (113.2ms in our example case) will be useful for instance in a case where a clinician measures the T_1_ in a new lesion of his patient at one time point *t*; say he gets a measure of *T*_1_(*t*) = *x* ms. If he re-measures it right after, there is 95% probability that *T*_1_(*t* + 30*min*) lies within *x* ± 113.2 ms. Now, if he wants to control the evolution of the lesion one year later and he measures *T*_1_(*t* + 1*year*) still within *x* ± 113.2 ms, he will not be able to say whether this change between *T*_1_(*t*) and *T*_1_(*t* + 1*year*) is due to an evolution of the tissue or to measurement errors.

**Table 1:** Repeatability indexes used to assess the repeatability of metrics over all WM according to vertebral levels.

	*Level*	*Mean ± SD*_*subjects*_	*CI*_*d*_	*ICC*	*MDC* *[% of SD*_*subjects*_*]*
***T***_***1***_ ***(ms)***	**C2**	964.9 ± 70.7	-17.2 to 66.0	0.46	± 158.4 [224.1]
	**C3**	1007.2 ± 74.3	-38.5 to 23.1	0.72	± 113.2 [152.3]
	**C4**	1060.0 ± 69.5	-63.6 to 4.8	0.53	± 135.5 [195.0]
	**C5**	1083.6 ± 95.2	-68.0 to 33.4	0.43	± 189.1 [198.7]
***MTR (%)***	**C2**	46.83 ± 1.52	-0.99 to 0.54	0.43	± 2.85 [186.7]
	**C3**	45.78 ± 1.38	-1.54 to 0.42	-0.3	± 3.76 [271.6]
	**C4**	44.87 ± 1.55	-1.14 to 0.75	0.16	± 3.53 [228.1]
	**C5**	44.02 ± 1.9	-2.0 to 0.66	0.05	± 5.06 [265.9]
***MT***_***sat***_ ***(%)***	**C2**	3.579 ± 0.194	-0.12 to 0.113	0.5	± 0.429 [220.6]
	**C3**	3.492 ± 0.184	-0.058 to 0.189	0.51	± 0.466 [253.6]
	**C4**	3.49 ± 0.21	-0.003 to 0.247	0.27	± 0.501 [238.1]
	**C5**	3.562 ± 0.266	-0.132 to 0.162	0.33	± 0.544 [204.9]
***MTV (%)***	**C2**	37.36 ± 2.38	-1.58 to 0.81	0.48	± 4.46 [187.4]
	**C3**	36.84 ± 2.55	-0.94 to 1.53	0.52	± 4.57 [178.8]
	**C4**	36.25 ± 2.44	-0.64 to 1.57	0.6	± 4.15 [169.8]
	**C5**	35.92 ± 2.5	-0.98 to 1.73	0.33	± 5.05 [202.2]

From left to right, the columns correspond to the mean ± SD across subjects (n = 16) based on values from the first scan session only, the 95% confidence interval for the true test-retest difference, the ICC coefficient, the MDC. All numbers are in the metric unit except those in square brackets, which are expressed as a percentage of the SD across subjects to quantify the repeatability relative to the inter-subject difference, i.e. the reliability. Fig 2 is a subset of this table.

**Table 2:** Repeatability indexes used to assess the repeatability of metrics in different sub-regions of the WM.

	*ROI*	*Mean ± SD*_*subjects*_	*CI*_*d*_	*ICC*	*MDC* *[% of SD*_*subjects*_*]*
***T***_***1***_ ***(ms)***	**WM**	1011.2 ± 60.8	-29.4 to 19.3	0.74	± 89.3 [146.8]
	**DC**	1068.3 ± 63.7	-69.0 to 5.4	0.41	± 146.8 [230.5]
	**LF**	971.6 ± 64.5	-22.9 to 39.6	0.52	± 115.6 [179.2]
	**VF**	1006.8 ± 168.0	-60.0 to 71.5	0.68	± 240.8 [143.4]
***MTR (%)***	**WM**	45.82 ± 1.3	-0.99 to 0.37	0.28	± 2.55 [196.2]
	**DC**	46.08 ± 0.87	-1.02 to 0.07	0.29	± 2.15 [247.3]
	**LF**	46.09 ± 1.54	-0.94 to 0.53	0.38	± 2.73 [178.1]
	**VF**	44.64 ± 2.48	-1.65 to 0.93	0.35	± 4.8 [193.1]
***MT***_***sat***_ ***(%)***	**WM**	3.517 ± 0.177	-0.022 to 0.152	0.6	± 0.34 [192.4]
	**DC**	3.452 ± 0.181	-0.029 to 0.25	0.1	± 0.543 [299.5]
	**LF**	3.59 ± 0.202	-0.009 to 0.118	0.82	± 0.252 [124.9]
	**VF**	3.438 ± 0.283	-0.124 to 0.172	0.54	± 0.546 [192.6]
***MTV (%)***	**WM**	36.79 ± 2.3	-0.96 to 1.13	0.6	± 3.83 [166.4]
	**DC**	36.46 ± 2.24	-0.65 to 1.34	0.6	± 3.7 [164.7]
	**LF**	36.88 ± 2.41	-1.21 to 0.89	0.65	± 3.87 [160.6]
	**VF**	37.17 ± 2.95	-1.2 to 1.81	0.42	± 5.58 [189.1]

From left to right, the columns correspond to the mean ± SD across subjects (n = 16) based on values from the first scan session only, the 95% confidence interval of the true test-retest difference, the ICC coefficient, the MDC. All numbers are in the metric unit except those in square brackets, which are expressed as a percentage of the SD across subjects to quantify the repeatability with respect to the inter-subject difference, i.e. the reliability. Fig 3 is a subset of this table.

**Fig 4:**
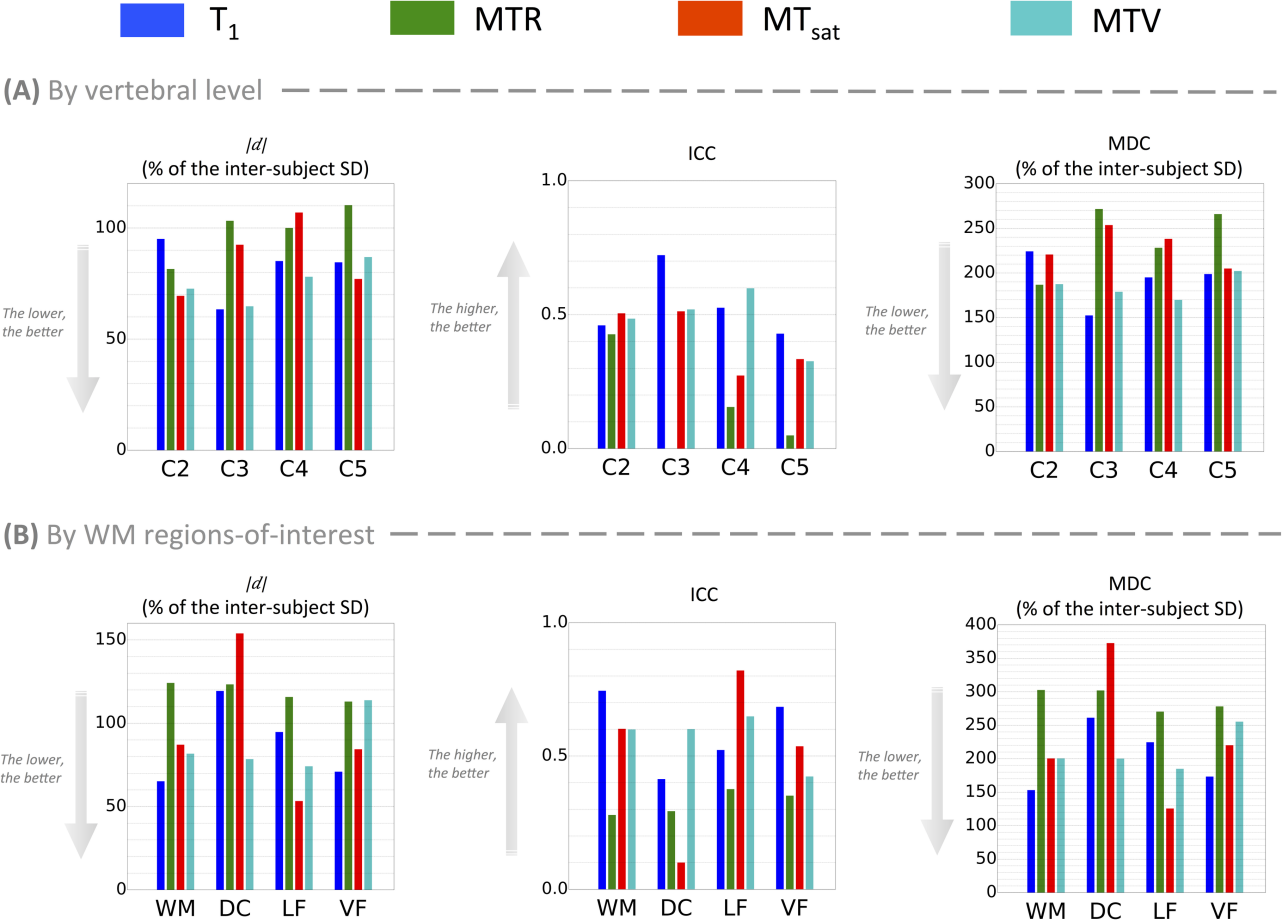
**Comparison between the repeatability of the four myelin-sensitive metrics when the metric is estimated (A) in the whole WM by vertebral level and (B) from C2 to C4 within WM sub-ROIs.** Repeatability indexes from left to right: mean absolute test-retest difference ($\bar{\left|d\right|}$), Intra-Class Correlation (ICC) coefficient, Minimal Detectable Change (MDC). $\bar{\left|d\right|}$ and MDC are expressed in percentage of inter-subject SD in order to assess the repeatability relative to the differentiation between subjects (i.e., the reliability), despite the different units of the metrics.

The ICC and the MDC (expressed in percentage of the SD across subjects) are useful to compare repeatability across metrics (more extensively done in Fig 4). For example, if we compare T_1_ to MTR at C3, the ICC is much higher for T_1_ (0.72) than MTR (-0.3)–note here that the interpretation of a negative value for the ICC is the same as for a null value (very poor reliability). This is because T_1_ has a lower test-retest variation (${\bar{\left|d\right|}}_{C3}$ = 47.1ms in Fig 2) compared to the variation between subjects (*SD*_*subjects*_ = 74.3ms in Table 1), whereas MTR has a high test-retest variation (${\bar{\left|d\right|}}_{C3}$ = 1.43% in Fig 2) compared to the variation between subjects (*SD*_*subjects*_ = 1.38% in Table 1). This also reflects in the MDC ($MDC=1.96\sqrt{2}\cdot {SD}_{total}\sqrt{1-ICC}$). For T_1_ at C3, MDC = 113.2ms, which is 152.3% of *SD*_*subjects*_ (Table 1), whereas for MTR at C2, MDC = 3.76%, which is 271.6% of *SD*_*subjects*_. This result shows that measurement errors in MTR cover almost 3 times the standard variations between subjects, making it difficult to observe true differences in MTR.

The mean test-retest difference ($\bar{\left|d\right|}$, displayed in gray at the top left of each graph) is higher at C5 (Fig 2); however, one-way repeated ANOVAs testing the effect of vertebral levels on the absolute test-retest difference did not report significant results (p-values were 0.183, 0.195, 0.389 and 0.579 for T_1_, MTR, MT_sat_ and MTV respectively). No clear test-retest difference between young and elderly subjects is observed on this graph.

For all metrics and all levels, no significant systematic bias between test and retest is detected (all *CI*_*d*_ include 0, see Table 1). When compared to other metrics, mean MT_sat_ shows minimal variations across vertebral levels (p-values of the repeated ANOVAs between levels were <<0.0001, <<0.0001, 0.02 and <0.0001 for T_1_, MTR, MT_sat_ and MTV respectively). The ICC coefficient highlights a poor test-retest reliability, barely exceeding 0.5, especially for MTR and MT_sat_. This point is supported by the MDC, which is generally around 2 times the SD across subjects.

Fig 3 shows repeatability results within sub-regions of the WM: dorsal column (DC), lateral funiculi (LF) and ventral funiculi (VF). Overall, the VF shows the largest test-retest differences. These observations were confirmed (except for MT_sat_ which shows large test-retest differences in the DC) by one-way repeated ANOVAs performed between ROIs on the absolute test-retest difference (p-values <0.01, 0.01, 0.08, <0.01 for T_1_, MTR, MT_sat_, MTV respectively). In addition, similar repeatability is found when the metrics are estimated over all WM or within the DC or the LF.

Fig 3 is a subset of Table 2, which quantifies the metrics repeatability within sub-ROIs of the WM from C2 to C4. Interestingly, MT_sat_ performs really differently according to the ROI, yielding the worst repeatability result in the DC (ICC = 0.1, MDC ≈ 3 inter-subject SDs) and the best one in the LF (ICC = 0.82, MDC ≈ 1.2 inter-subject SDs). Note however that estimating the metric at several levels (here, C2 to C4) is not favorable to MT_sat_ given that its ICC in WM at C4 is half its ICC at C3 (Table 1). Overall, T_1_ and MTV yield the best results. MTV regularly shows a fair repeatability whatever the ROI is, with a MDC about 1.5 to 2 times the inter-subject SD (which is equivalent to 87–95% of the sample distribution). In the level-wise analysis, MTV performs slightly better than T_1_. We suspect that these results reflect the clearer delineation between the cord sub-regions and the more homogeneous values in those sub-regions that could be observed in MTV maps when compared to T_1_ or even MT_sat_ maps (Fig 1). Furthermore, as expected, MTR regularly performs worst, in part because of the low contrast between subjects it exhibits, whatever the ROI is.

Fig 4 compares three main repeatability indexes (absolute test-retest difference, ICC and MDC) between the different metrics. While no particular metric stands out from this comparison, MTR seems to be the least reliable at every level. For most of the vertebral levels, $\bar{\left|d\right|}$ of MTR is on the same order as the inter-subject SD (which is equivalent to 68% of the population if we assume a Normal distribution for the sample), the ICC is below 0.4 at every level and the MDC exceeds 2.5 inter-subjects SDs (equivalent to 98.8% of the population) at 2 levels over 4. When considering the effect of vertebral level, C5 seems to be the least reliable (ICC < 0.5 for all metrics). Regarding the effect of WM regions (Fig 4B), some differences are observed. For instance, MT_sat_ yields the best ICC score in the LF (0.82) and the worst in the DC (0.1).

### 3.2. Sensitivity to myelin content

This section deals with the larger sample (n = 33 subjects).

#### 3.2.1. Effects of vertebral levels and WM regions

**Fig 5:**
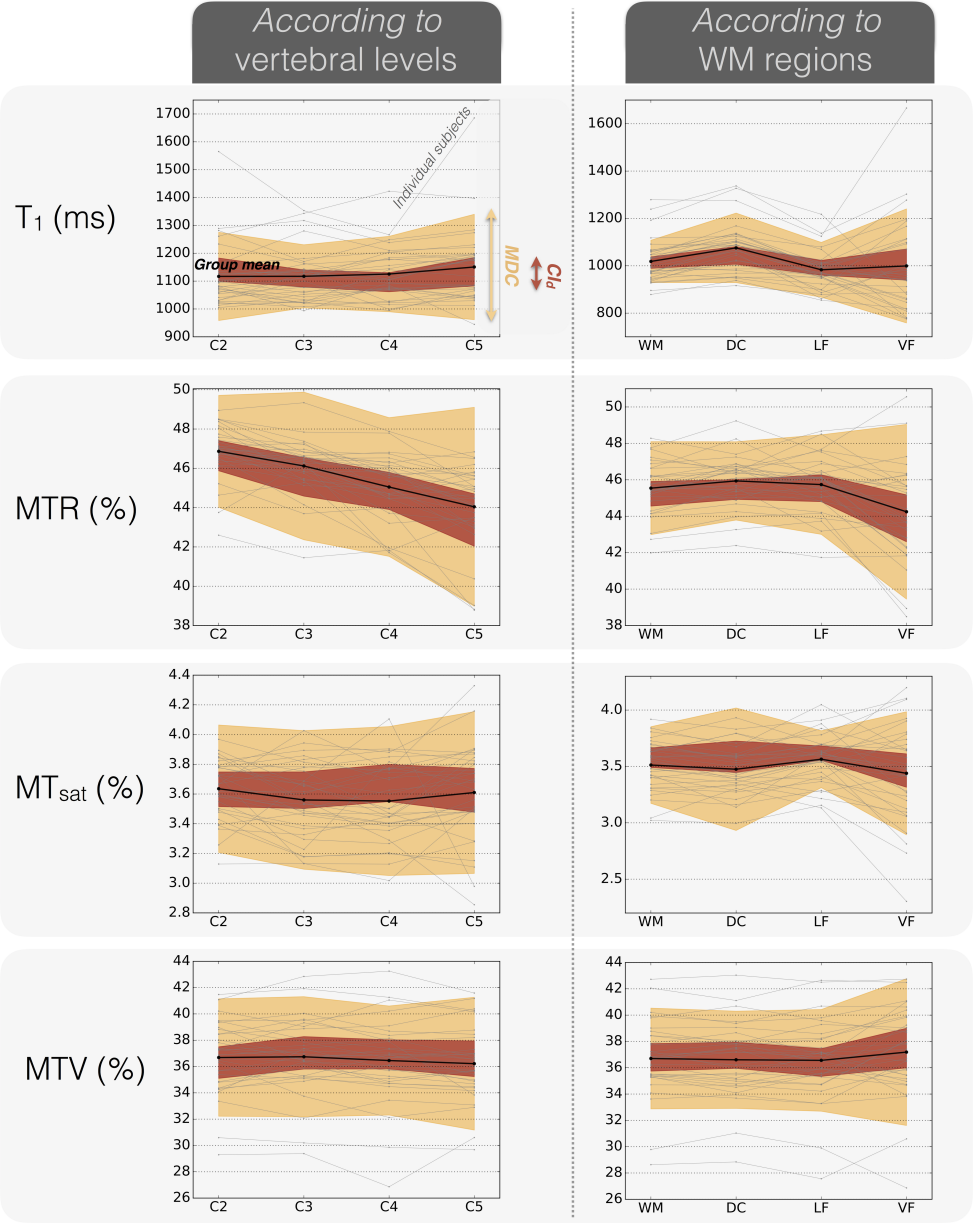
Comparison across vertebral levels and WM regions along with the measurement errors for the group mean (n = 33) and individual subjects. The red envelope represents the 95% confidence interval for the test-retest difference (CI_d_), which assesses the measurement error magnitude of the group mean (in black). The orange envelope represents the MDC (Minimum Detectable Change), difference required to compare individual subjects (faded gray lines). Note that the group mean approaching the edges of the CI_d_ (red envelope) reflects an asymmetric confidence interval due to a non-null offset between test and retest (non-null mean test-retest difference, $\bar{d}$). However, no offset was large enough to report a significant systematic bias between test and retest (see section 3.1. Repeatability, Table 1 and Table 2).

Fig 5 plots the group mean along with the measurement error magnitude (*CI*_*d*_) in order to allow the reader to assess whether differences between vertebral levels or WM regions can be distinguished from measurement errors or not. Individual subjects data are also plotted to see if differences between subjects can be carried out despite the measurement error. However, for individual comparison, measurement errors are assessed by the MDC, which is much larger than the *CI*_*d*_ (as negative and positive errors do not compensate for each other). Only T_1_ and MTV seem to allow the comparison between some healthy subjects.

The differences that are distinguishable from measurement errors were sum up in Table 3, along with the results of the one-way repeated ANOVAs. One can observe that some cases show significant differences but those differences are too small to be distinguished from measurement errors. This is the case for the MTR which is significantly different between every vertebral level but only C2 and C5 show a difference large enough to be due to something else than measurement errors. Also, significant differences between WM regions are found with MTR and T_1_ but none of them are larger than measurement errors.

**Table 3:** Comparison of significantly different vertebral levels (A) or WM regions (B) with differences larger than measurement errors.

	(A) Analysis by vertebral levels			(B) Analysis by WM regions		
	*p*	*Significantly different levels (p<0*.*05)*	*Differences larger than measurement errors*	*p*	*Significantly different ROIs (p<0*.*05)*	*Differences larger than measurement errors*
**T**_**1**_ **(ms)**	0.041	• C2 vs. C5	None.	<0.01	• DC vs. LF • DC vs. VF	None.
**MTR (%)**	<<10^−4^	• All levels are significantly different from each other.	• C2 vs. C5	<<10^−4^	• DC vs. VF • LF vs. VF	None.
**MT**_**sat**_ **(%)**	0.189		None.	0.076		None.
**MTV (%)**	0.081		None.	0.085		None.

For each analysis (A, B), the left column is the results of the one-way repeated ANOVAs whereas the right column reports the vertebral levels/WM regions showing differences larger than measurement errors (see also Fig 5).

#### 3.2.2. Effect of age

**Fig 6:**
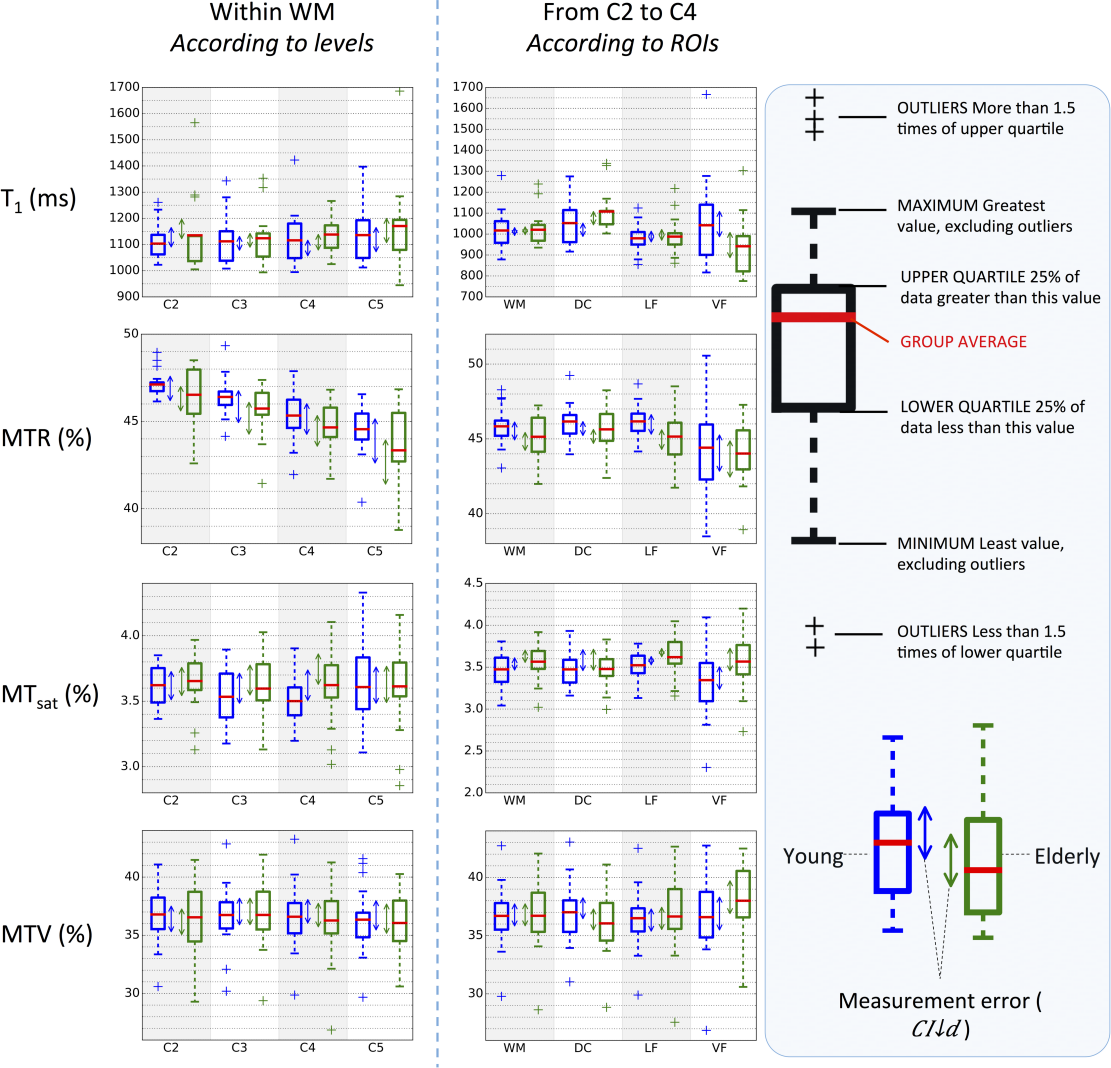
Comparison between young (n_young_ = 19) and elderly (n_elderly_ = 14) subjects along with measurement errors. For each case, the corresponding 95% confidence interval for the mean test-retest difference (*CI*_*d*_), estimated from the test-retest analysis (see section 3.1. Repeatability) was centered at the mean of each group, in order to assess whether the difference between young and elderly is larger than the test-retest errors or not. With all metrics within every spinal cord region (vertebral level or WM region), the difference in means between young and elderly was undistinguishable from measurement errors.

Fig 6 compares the differences between young and elderly to the measurement errors assessed by the *CI*_*d*_. With all metrics within every spinal cord region (vertebral level or WM region), the difference between young and elderly can always be explained by measurement errors only. Moreover, the repeated ANOVAs did not report any significant effect of age for all metrics, neither level-wise nor ROI-wise. However, we can still notice some general trends: T_1_, MTR and MTV generally support the demyelination with aging histologically reported in the literature, whereas MT_sat_ constantly shows the reverse trend.

To complete this study, Table 4 reports the statistical power analysis. From this analysis, one can compare the difference that can be detected given the metrics test-retest errors (length of the *CI*_*d*_, 2^nd^ column) to the minimum difference in the true metric values required to detect a significant difference (1^st^ column) between young and elderly (with a fair test power). We can notice for example that, given the measurement errors of MTR (1.36%), even if the difference in means were large enough (≥1.27%) to yield a significant result, the imprecision of measurement is too large to detect such a difference. It is not the case with the other metrics. Moreover, we can notice that the observed differences in means (3^rd^ column) are very low compared to the difference needed to obtain significant results (1^st^ column), yielding very low statistical power for those tests (4^th^ column). Finally, given the large sample size required to obtain a significant difference (5^th^ column), T_1_ and MTV do not seem sensitive to age groups (based on their mean WM values in this study).

**Table 4:** Power analysis based on each metric WM values for a two-sample t-test between young and elderly subjects with a significance level of 5%.

	Minimum difference required to detect a significant difference with such a sample and 80% probability (effect size)	Length of *CI*_*d*_	Observed difference in means (*young* – *elderly*)	Power (probability to detect a significant difference with such a sample)	Sample size required to detect a significant difference with such means and 80% probability
***T***_***1***_ ***(ms)***	93.8	48.7	-3.6	5.1%	10394
***MTR (%)***	1.27	1.36	0.70	33.5%	52
***MT***_***sat***_ ***(%)***	0.203	0.174	-0.092	24.5%	75
***MTV (%)***	2.67	2.09	-0.01	5.0%	1690133

## 4. Discussion

This study proposes a statistical framework for comparing clinically feasible myelin imaging techniques (T_1_, MTR, MT_sat_ and MTV) in the cervical spinal cord.

### 4.1. Myelin-sensitive metrics values in the spinal cord

The resulting mean values across subjects are in agreement with previous studies. Stikov et al. [68] observed a T_1_ around 1000ms in the brain, which is comparable to the T_1_ in the spinal cord WM *in-vivo* at 3T [69,70]. The same holds for our MTV measurements which are in agreement with reported PD values [12,18–23,69,71]. There is no gold-standard for clinically feasible MT-based protocols due to their dependence on pulse sequence parameters. However, the values for MTR and MT_sat_ we observed are also in agreement with literature [6,45,48,72–75].

### 4.2. Repeatability

Even for the most reliable metrics (T_1_ and MTV, see Fig 4), the ICC is moderate (around 0.5) and the MDC is on the order of two inter-subject SDs. Given the test-retest variations, the minimal difference between individual healthy subjects that can be detected with these metrics (MDC) is much larger than the usual variations we observed (see Fig 5). Looking at groups of subjects, significant differences between spinal cord regions stand out but still, they are not large enough to be distinguished from measurement errors (quantified by the *CI*_*d*_ in this case, as shown in Fig 5).

In comparison with the brain, repeatability in the spinal cord is hampered by multiple sources of artifacts (motion, susceptibility) and low SNR [43]. Better repeatability might be achieved with coarser resolution and/or more averaging, though at the cost of longer acquisition times, which could be associated with more subject motion.

Taso et al. [44] reported results for myelin-related metrics in the spinal cord WM: a CV of 5.3% for MTR and 2.9% for ihMT ratio. However, this study reported the repeatability in terms of CVs, which are misleading when comparing metrics with different units and/or dynamic ranges (as mentioned in section 2.3.1. Repeatability). Smith et al. [48] reported a *CI*_*d*_ of [− 3%, +5%] for MTR over all WM from C2 to C5 within 10 young healthy subjects. Even if the repeatability of the metrics reported in our study is not good enough to differentiate between WM regions or age groups, it is still much better (*CI*_*d*_ of [− 0.99%, +0.54%] for MTR). This may suggest that significant differences not accounting for precision of measurements might have been reported in the literature, whereas they could be only explained by measurement errors.

Looking at the metrics individually, T_1_-based metrics (MTV and T_1_) generally show the best reliability (Fig 4). Regarding sensitivity to myelin, MTV shows clearer delineation of the GM and smooth variations in the WM (Fig 1), but no difference between WM regions stood out when compared to the measurement error. When looking at individual maps, T_1_ seems particularly affected by cord movements and compressions occurring during respiratory and cardiac cycles (Fig 1), which produces statistically significant differences (see Table 3), but those differences are not larger than measurement errors. The same applies for MTR, which emerges as the less reliable metric due to its very small variation between subjects (Fig 4). However, MTR is the only metric exhibiting a significant effect that accounts for measurement error (difference between vertebral levels C2 and C5 in Table 3). This decrease in MTR towards lower levels could reflect a true decrease in myelin content, but could also be due to B_1_^+^ inhomogeneity. MTR variations due to B_1_ errors have already been reported in the brain [76] and correcting for them should be further investigated in the spinal cord. MT_sat_ minimizes the T_1_ contribution included in MTR, and is thereby less variable across vertebral levels.

### 4.3. Sensitivity and specificity to myelin with MRI

The assessment of the sensitivity of metrics to myelin content remains difficult, due to the lack of a ground truth. A loss of myelinated fibers with aging (mainly the small caliber ones) was observed histologically in the brain [77] and cervical spinal cord [64–66] but it remains unclear if these variations can be detected by clinical MRI nowadays. Age effects have been reported in the brain with MTR [78] and DTI [79–82]. In the spinal cord, most age effects are reported with DTI [83–85]. One study investigated MTR evolution in the spinal cord during aging, but no significant effect was reported [44]. The same study reported a decrease in ihMT ratio between subjects aged 35 to 50 and subjects aged over 50, not accounting for measurement errors however. Our study did not observe any difference between age groups, with or without accounting for measurement error (Fig 6). This lack of sensitivity to aging could be due to the choice of acquisition parameters, the small effect/sample size, or simply due to a lack of true differences in myelination.

As noted in the introduction, some of the myelin-sensitive techniques are also hampered by confounding factors. For example, T_2_* is affected by iron content, fiber orientation, blood vessels and blood oxygen level. MTR is affected by T_1_ and B_1_ field, and more generally, magnetization transfer and MTV are sensitive to macromolecules (i.e., not only myelin). For each of these techniques, there are ways to mitigate those confounds. For example, quantitative susceptibility maps could inform T_2_* maps, or T_1_ and B_1_^+^ fields could be acquired to correct MTR maps [76]. All these strategies come at the cost of additional scan time, and possibly larger output variance (due to the introduction of yet other noisy measures).

While DTI has some intrinsic limitations, other techniques also based on diffusion-weighted imaging might offer more sensitivity to myelin. It is important to note, however, that because water protons trapped between myelin sheaths have a short T_2_ (around 10 ms at 3T, which could be quantified using myelin water fraction techniques) and that protons from bound molecules have an even shorter T_2_ (order of μs, which could be quantified with ultra-short TE imaging or magnetization transfer techniques), diffusion-weighted protocols typically use a TE (> 60ms) too long to be sensitive to signal coming from the myelin (and from water trapped in it). Some advanced diffusion-weighted techniques include NODDI [47,86], which can notably estimate the intra-cellular volume fraction and CHARMED/AxCaliber [87–89], which can notably estimate the hindered (extra-cellular) and restricted (intra-cellular) water fraction. All these metrics are thus indirectly related to the myelin volume fraction, although additional information would be required to be able to quantify absolute myelin content.

To improve specificity to myelin, combining several metrics, using for example independent component analysis, or acquiring maps of confounding factors for a posteriori corrections, might be advisable [90]. Future work will be undertaken in this direction [91].

### 4.4. Perspective of repeatability assessment

Repeatability assessment is crucial for the development of qMRI biomarkers. Our results show that significant differences between groups can be reported with standard statistical tests, yet these differences are comparable to (or even smaller than) test-retest measurement errors. Controlling for both aspects (statistical significance and measurement errors) is necessary for qMRI studies.

The indexes reported in this work (95% confidence interval for the test-retest difference (*CI*_*d*_), ICC and MDC) are useful for quantifying repeatability and allowing comparisons across studies. As mentioned before, the coefficient of variation depends on the magnitude of the metric, and should not be the primary index for assessing repeatability, especially if metrics have different means or units. The *CI*_*d*_ first allows to control for the existence of a potential systematic bias between measurements (i.e. scan sessions). In addition, it gives an estimation of the measurement error for group averages. In the same vein, the MDC provides a measure of the minimum difference between two individual measurements to report a true difference, taking into account the measurement errors. For example, the *CI*_*d*_ would be useful for researchers comparing different populations, whereas the MDC would be useful for a clinician needing to assess the evolution of a WM lesion within a single patient. Furthermore, the ICC coefficient has the advantage to be dimensionless, and can thus be easily compared to assess reliability across metrics, studies, vendors or sites. Aside from providing a robust quantification of the repeatability with two measurements (test-retest studies), the ICC coefficient (and consequently, the MDC) can also be consistently used with more than two measurements. Those reliability indexes have already been extensively used in test-retest studies from other research fields, such as rehabilitation, where the precision of tests is crucial [49–53]. In this work, the absolute test-retest difference (|*d*|) was reported to provide the reader with a direct and basic measure of measurement errors; however, this index is not sufficient to estimate the repeatability and compare it across studies.

Finally, the assessment of the repeatability needs to be adapted to the study goals. Indeed, the ICC depends on the sample homogeneity. Therefore, if the goal is to differentiate between the microstructure of healthy subjects, including patients in the sample will artificially increase the between-subjects variability and overestimate the ICC. In this study, we can confidently assert that the ICC is lower (and the MDC is higher) than it would have been for a sample that includes patients and controls. Therefore, if the goal is to distinguish between pathological cases, we recommend including the different types of tissue (healthy and pathological tissues, with different stages of the disease) in the cohort. This way, the MDC and ICC would integrate the associated between-subjects variability.

### 4.5. Data sharing

Due to IRB restrictions, all data used here could not be publicly shared. However, we obtained specific consent for sharing MRI data from four young volunteers. Three of them were part of the tested and retested group. Along with those datasets, we provide the batch scripts used to produce the myelin-sensitive metric maps and to register them to spinal cord template and white matter atlas. Also available is a Microsoft Excel spreadsheet gathering all results of the metric estimations within each region of interest for every scan session and every volunteer of the cohort. The 1^st^ tab of the sheet corresponds to the tested and retested cohort only (n = 16), and the 2^nd^ tab corresponds to the whole cohort (n = 33). Finally, also shared are the scripts to extract these metrics values, to compute the statistical indices for reliability assessment and to produce the figures presented in this work. All these data and code are available at: https://osf.io/ezmrj/.

## 5. Conclusion

In this study, we assessed the repeatability and distribution of myelin-sensitive metrics (T_1_, MTR, MT_sat_ and MTV) in the spinal cord. T_1_ and MTV (1 – *proton density*) showed the best reliability regarding the inter-subject variations, but the measurement error remains too large to detect differences between healthy individuals. T_1_, MTR and MTV showed trends consistent with the hypothesis of demyelination with aging, but again the differences were not large enough to be distinguishable from measurement errors, or to be significant.

This study used a range of statistical tools to explore the differences between myelin-sensitive metrics. We show that even though statistically significant differences can be reported using standard statistical tests, an important proportion of these differences can be attributed to measurement error. In particular, the coefficient of variation is a misleading index when comparing metrics with different units, and we recommend using the MDC when comparing individual measurements, and the 95% confidence interval of the test-retest difference when comparing groups. The indexes explored in this study allow for a fair comparison of qMRI metrics across studies, MRI vendors and sites, leading toward standardizing the field of myelin imaging and increasing its clinical relevance.

## Supporting information

S1 File Data processing pipeline. This section describes the data processing steps performed to estimate MTR, MT_sat_, T_1_ and MTV maps and to register those maps to the MNI-Poly-AMU template [55] and WM atlas [56].
(PDF)
