# Correction: Test-retest reliability of myelin imaging in the human spinal cord: Measurement errors versus region- and aging-induced variations

**DOI:** 10.1371/journal.pone.0199796

**Published:** 2018-06-28

**Authors:** Simon Lévy, Marie-Claude Guertin, Ali Khatibi, Aviv Mezer, Kristina Martinu, Jen-I Chen, Nikola Stikov, Pierre Rainville, Julien Cohen-Adad

There are errors in references 31, 39, 40, 54, 71, and 91. Please see the corrected references here:

31 Ljungberg IV, Emil, Tam R, Rauscher A, Li D, Traboulsee A, MacKay A, et al. Rapid Myelin Water Imaging in Human Cervical Spinal Cord. ISMRM 24th Annual Meeting & Exhibition. Singapore; 2016.
39 Alsop DC, de Bazelaire C, Garcia DM, Duhamel G. Inhomogenous magnetization transfer imaging: a potentially specific marker for myelin. 13th Annual Meeting of ISMRM. Miami, Florida, USA; 2005. p. 2224.
40 Alsop DC, Dandamudi R, Bakshi R. Inhomogeneous magnetization transfer imaging of myelin concentration in multiple sclerosis. 15th Annual Meeting of ISMRM. Berlin, Germany; 2007. p. 2188.
54 De Leener B, Lévy S, Dupont SM, Fonov VS, Stikov N, Louis Collins D, et al. SCT: Spinal Cord Toolbox, an open-source software for processing spinal cord MRI data. NeuroImage. 2017;145: 24–43. doi:10.1016/j.neuroimage.2016.10.009
71 Duval T, Lévy S, Stikov N, Campbell J, Mezer A, Witzel T, et al. g-Ratio weighted imaging of the human spinal cord in vivo. NeuroImage. 2017;145, Part A: 11–23. doi:http://dx.doi.org/10.1016/j.neuroimage.2016.09.018
91 Lévy S, Khatibi A, Mangeat G, Chen J-I, Martinu K, Rainville P, et al. Statistical combinations of T1, MTR, MTsat and Macromolecular Tissue Volume to improve myelin content estimation in the human spinal cord at 3T. ISMRM 25th Annual Meeting & Exhibition. Honolulu, Hawai, USA; 2017.
