# Supplementary material for: Test-retest reliability of myelin imaging in the human spinal cord: Measurement errors versus region- and aging-induced variations
Source: PLoS One. 2018 Jan 2;13(1):e0189944. doi: 10.1371/journal.pone.0189944 (PMC5749716; doi:10.1371/journal.pone.0189944)
Supplement: S1 File — This section describes the data processing steps performed to estimate MTR, MTsat, T1 and MTV maps and to register those maps to the MNI-Poly-AMU template [55] and WM atlas [56]. (PDF) [file pone.0189944.s001.pdf]

Test-retest reliability of myelin imaging in the human spinal cord: measurement errors versus region- and aging-induced variations

Simon Lévy[1,3,#a], Marie-Claude Guertin[2], Ali Khatibi[3,4,5,6,#b], Aviv Mezer[7], Kristina Martinu[3], Jen-I Chen[3,8], Nikola Stikov[1,9], Pierre Rainville[3,8], Julien Cohen-Adad[1,10]*

[1]NeuroPoly Lab, Institute of Biomedical Engineering, Polytechnique Montreal, Montreal, QC, Canada

[2]Montreal Health Innovations Coordinating Center (MHICC), Montreal Heart Institute, Montreal, QC, Canada.

[3]Centre de Recherche de l'Institut Universitaire de Gériatrie de Montréal (CRIUGM), Montréal, QC, Canada

[4]Psychology Department, Bilkent University, Ankara-06800, Turkey

[5]Interdisciplinary program in Neuroscience, Bilkent University, Ankara-06800, Turkey

[6]National Magnetic Resonance Research Center (UMRAM), Bilkent University, Ankara-06800, Turkey

[7]The Edmond and Lily Safra Center for Brain Sciences (ELSC), The Hebrew University of Jerusalem, Jerusalem, Israel

[8]Department of Stomatology, Faculty of Dentistry, Université de Montréal, Montreal, QC, Canada

[9]Montreal Heart Institute, Montreal, QC, Canada

[10]Functional Neuroimaging Unit, CRIUGM, Université de Montréal, Montreal, QC, Canada

[#a]Current Address: Centre d'Exploration Métabolique par Résonance Magnétique (CEMEREM), AP-HM, Hôpital de la Timone, Pôle d'imagerie médicale, Marseille, France

[#b]Current Address: Psychology Department, Bilkent University, Ankara-06800, Turkey

*Corresponding author
Email: jcohen@polymtl.ca

Supplementary material

This section describes the data processing steps performed to estimate MTR, MT_{sat}, T_1 and MTV maps (see introduction section in the main manuscript for definitions) and to register those maps to the MNI-Poly-AMU template [1] and WM atlas [2].

1. Metric calculation

Analysis was performed using the *Spinal Cord Toolbox* (SCT) version 2.2.3 [3]. The four FLASH images were first corrected for motion as follows: the image with flip angle 20° was used as target for the three other images which were first registered using translations constrained in the z direction (2 degrees of freedom) and then slice-by-slice regularized translations [4]. Each metric was then computed as follows.

1.1. MTR

The MTR (%) map was computed voxel-wise using the 3D FLASH images with 10° flip angle with (MT_{on}) and without (MT_{off}) off-resonance saturation pulse according to the formula:

$$MTR = \frac{MT_{off} - MT_{on}}{MT_{off}} \times 100$$

1.2. MT_{sat}

The MT_{sat} map was computed according to the method described in Helms et al. [5] using the 3D FLASH image with 10° flip angle and off-resonance saturation pulse for the MT-weighted image, and the 3D FLASH images (without off-resonance saturation) with 4 and 20° flip angles as the PD- and T_1-weighted images respectively.

1.3. T_1

The T_1 map was obtained using the Variable Flip Angle (VFA) method, analytically resolving the steady-state signal of the FLASH sequence according to the exact solution derived in Ganter [6]. This method accounts for incomplete spoiling and was shown to reduce the usual T_1 overestimation of the VFA method [7] when compared to the gold-standard inversion recovery method in the brain WM [8]. Briefly, it models the steady-state signal as:

$$S(\alpha) = K \cdot \left| -i \frac{1 - E_1}{D} \sin(\alpha) \cdot (1 - \Lambda^*) \right| \, with \, E_1 = \exp\left(-\frac{TR}{T_1}\right)$$

D is a function of the flip angle α, E_1 and Λ, while Λ is defined by a continued fraction expansion [6].

The flip angle α was corrected for transmit field inhomogeneities by computing a B_1^+ map. This map was obtained using the double angle method [9-11] based on the 2D segmented spin-echo EPI with flip angles of 60° and 120°; a strong smoothing (kernel of 25x25 voxels) was finally applied in plane in order to account for the low frequency profile of the transmit field.

Λ depends on T_2. Therefore prior knowledge of the T_2 value is necessary to solve the equation. As the T_2 values of WM and GM are relatively close, a value of 73 ms was used for the whole cord. For CSF, a T_2 value of 2500 ms was used [12].

1.4. PD and MTV

In the previous equation, K is a proportionality factor that includes the proton density, the coil gain and the T_2^* relaxation time; it can be modeled as $K = M0 \cdot \exp\left(-\frac{TE}{T_2^*}\right)$ where $M0 = g \cdot PD$ with g the coil gain and PD the water proton density. Thus, once T_1 and K are determined, we can obtain $M0$ using T_2^* values of 50 and 120 ms for the cord and CSF respectively [13,14] (T_2^* relaxation has a very small impact on the determination of $M0$ given the very short TE). Then, we use the $M0$ estimate to derive g and PD. To do so, the method of analytical estimation of g [15] was adapted to the spinal cord. This method consists of 5 general steps:

1- Splitting the cord into 2 clusters (WM and GM) using a *k*-means clustering algorithm based on T_1 values,

2- Parceling the region of interest (ROI) into 3D boxes of 10x10x50 mm with a 50% overlap between adjacent boxes,

3- Discarding the boxes that do not include enough voxels (according to the clustering made at step #1),

4- In each box, repeating the following steps six times:

a. Estimating PD based on the T$_1$ map and the empirical relation $\frac{1}{PD} = A + \frac{B}{T_1}$ [15-17] with initial values for the constants A and B taken from the literature (0.916 and 436 respectively),

b. Estimating g as $g = M0/PD$,

c. Smoothing the estimated g by fitting a 3D 3rd order polynomial basis,

d. Calculating the new value of the constants A and B with the new g and the new $PD = \frac{M0}{g}$.

5- Calculating a global gain from the local gains (g_i) estimated in each box:

a. Calculating a scale factor ($f_{i,j}$) between adjacent boxes i and j in the overlapping regions by resolving the multilinear system of minimization equations:

$$\min_{f_{i,j}}\{|g_i - g_j \cdot f_{i,j}|\} \; for\ every\ i,j \in [1,2,\dots,n]\ and\ i \neq j$$

b. Joining all the g_i using the scale factors calculated previously to get a global gain (g) in the cord,

c. Fitting a 3D 3rd order polynomial basis to g in order to get an estimation of the global gain in the whole image.

Finally, the PD needs to be calibrated by its value in CSF, assuming that CSF is only made of liquid protons. To do so, the peak of the voxel values distribution in the CSF was used to normalize the PD map: $PD := \frac{PD}{PD_{CSF}}$. The MTV is defined as the complement of the free proton density, i.e. $MTV = 1 - PD$.

2. Template registration and metric estimation

For metric estimation within ROIs, the MNI-Poly-AMU template [1] and WM atlas [2] were registered to the FLASH images. To do so, the warping field $\mathrm{WF}_{\mathrm{template} \rightarrow \mathrm{anat}}$ from the template to the sagittal anatomic image, which shows a high contrast between cord and CSF, was first estimated by registering the template to the sagittal image. Then, the warping field $\mathrm{WF}_{\mathrm{anat} \rightarrow \mathrm{FLASH}}$ from the sagittal anatomic image to the FLASH images was estimated based on the template WM probabilistic map (registered to the sagittal image) and the GM segmentation made on the MT-weighted FLASH image (MT_{on}), allowing us to take into account the subject's GM shape. Finally, the warping fields $\mathrm{WF}_{\mathrm{template} \rightarrow \mathrm{anat}}$ and $\mathrm{WF}_{\mathrm{anat} \rightarrow \mathrm{FLASH}}$ were concatenated to get a global warping field $\mathrm{WF}_{\mathrm{template} \rightarrow \mathrm{FLASH}}$ from the template space to the FLASH images space. Applying this global warping field to the WM atlas enables to get a fairly good estimation (robust to noise and partial volume effects) of each metric value in the different WM pathways using a *Maximum A Posteriori* approach, as proposed in Lévy et al. [2].

To study the metrics variations across WM regions, metrics were estimated within three sub-regions of the WM (right and left gathered) based on the spinal cord WM atlas proposed by Lévy et al. [2]:

- The dorsal column (DC): fasciculus gracilis, fasciculus cuneatus;
- The lateral funiculi (LF): lateral corticospinal tract, spinocerebellar tract, rubrospinal tract, lateral reticulospinal tract, spinal lemniscus;
- The ventral funiculi (VF): spino-olivary tract, ventrolateral reticulospinal tract, lateral vestibulospinal tract, ventral reticulospinal, ventral corticospinal tract, tectospinal tract, medial reticulospinal tract, medial longitudinal fasciculus.

3. References

1. Fonov VS, Le Troter A, Taso M, De Leener B, Lévêque G, Benhamou M, et al. (2014) Framework for integrated MRI average of the spinal cord white and gray matter: The MNI–Poly–AMU template. NeuroImage 102, Part 2: 817-827.

2. Lévy S, Benhamou M, Naaman C, Rainville P, Callot V, Cohen-Adad J (2015) White matter atlas of the human spinal cord with estimation of partial volume effect. NeuroImage 119: 262-271.

3. De Leener B, Lévy S, Dupont SM, Fonov VS, Stikov N, Louis Collins D, et al. (2016) SCT: Spinal Cord Toolbox, an open-source software for processing spinal cord MRI data. NeuroImage.

4. Cohen-Adad J, Lévy S, Avants B. Slice-by-slice regularized registration for spinal cord MRI: SliceReg 2015; Toronto, Canada.

5. Helms G, Dathe H, Kallenberg K, Dechent P (2008) High-resolution maps of magnetization transfer with inherent correction for RF inhomogeneity and T1 relaxation obtained from 3D FLASH MRI. Magnetic Resonance in Medicine 60: 1396-1407.

6. Ganter C (2006) Steady state of gradient echo sequences with radiofrequency phase cycling: Analytical solution, contrast enhancement with partial spoiling. Magnetic Resonance in Medicine 55: 98-107.

7. Stikov N, Boudreau M, Levesque IR, Tardif CL, Barral JK, Pike GB (2015) On the accuracy of T1 mapping: Searching for common ground. Magnetic Resonance in Medicine 73: 514-522.

8. Heule R, Ganter C, Bieri O (2015) Variable flip angle T1 mapping in the human brain with reduced t2 sensitivity using fast radiofrequency-spoiled gradient echo imaging. Magnetic Resonance in Medicine: n/a-n/a.

9. Insko E, Bolinger L. B1 mapping; 1992. pp. 4302.

10. Stollberger R, Wach P (1996) Imaging of the active B1 field in vivo. Magnetic Resonance in Medicine 35: 246-251.

11. Boudreau M, Tardif CL, Stikov N, Sled JG, Wayne L (2017) B1 Mapping for Bias-correction in Quantitative T1 Imaging of the Brain at 3 Tesla Using Standard Pulse Sequences. Journal of Magnetic Resonance Imaging.

12. Smith SA, Edden RAE, Farrell JAD, Barker PB, Van Zijl PCM (2008) Measurement of T1 and T2 in the cervical spinal cord at 3 tesla. Magnetic Resonance in Medicine 60: 213-219.

13. Wansapura JP, Holland SK, Dunn RS, Ball WS (1999) NMR relaxation times in the human brain at 3.0 tesla. Journal of Magnetic Resonance Imaging 9: 531-538.

14. Péran P, Hagberg G, Luccichenti G, Cherubini A, Brainovich V, Celsis P, et al. (2007) Voxel-based analysis of R2* maps in the healthy human brain. Journal of Magnetic Resonance Imaging 26: 1413-1420.

15. Mezer A, Yeatman JD, Stikov N, Kay KN, Cho N-J, Dougherty RF, et al. (2013) Quantifying the local tissue volume and composition in individual brains with magnetic resonance imaging. Nat Med 19: 1667-1672.

16. Gelman N, Ewing JR, Gorell JM, Spickler EM, Solomon EG (2001) Interregional variation of longitudinal relaxation rates in human brain at 3.0 T: Relation to estimated iron and water contents. Magnetic Resonance in Medicine 45: 71-79.

17. Panos P. Fatouros, Anthony Marmarou (1999) Use of magnetic resonance imaging for in vivo measurements of water content in human brain: method and normal values. Journal of Neurosurgery 90: 109-115.
